# Real-World Clinical Outcomes of Ribociclib in Combination with a Non-Steroidal Aromatase Inhibitor and a Luteinizing Hormone-Releasing Hormone Agonist in Premenopausal HR+/HER2− Advanced Breast Cancer Patients: An Italian Managed Access Program

**DOI:** 10.3390/curroncol29090521

**Published:** 2022-09-17

**Authors:** Nicoletta Staropoli, Elena Geuna, Gaetana Rinaldi, Giancarlo Bisagni, Vieri Scotti, Giovanni Faggioni, Laura Vannini, Carlo Arcara, Gabriella Moretti, Marco Gunnellini, Luigi Coltelli, Francesco Verderame, Lorenzo Livi, Giuseppina Sanna, Donatella Grasso, Giulia Abbinante, Francesca Ragni

**Affiliations:** 1 Medical Oncology and Translational Medical Oncology Units, Department of Experimental and Clinical Medicine, Magna Graecia University, AOU Materdomini Catanzaro, Campus Salvatore Venuta, 88100 Catanzaro, Italy; 2 Multidisciplinary Oncology Outpatient Clinic, Candiolo Cancer Institute, FPO-IRCCS, 10060 Candiolo, Italy; 3 Department of Surgical, Oncological and Oral Sciences, Section of Medical Oncology, University of Palermo, 90133 Palermo, Italy; 4 Department of Oncology, Azienda Sanitaria Universitaria Friuli Centrale, 33100 Udine, Italy; 5 Department of Oncology, Radiation Therapy Unit, Careggi University Hospital, 50134 Florence, Italy; 6 Medical Oncology 2, Istituto Oncologico Veneto IRCCS, 35128 Padova, Italy; 7 Department of Oncology, University of Florence, 50134 Florence, Italy; 8 Medical Oncology Unit, La Maddalena Hospital, 90146 Palermo, Italy; 9 Oncologia Medica USL Umbria 1, Gubbio, 06024 Perugia, Italy; 10 UOC Oncologia Ospedale Civile Livorno USL Toscana Nord Ovest Livorno, 57124 Livorno, Italy; 11 Villa Sofia-Cervello Hospital, 90146 Palermo, Italy; 12 Sandro Pitigliani’ Medical Oncology Department, Istituto Toscano Tumori, 59100 Prato, Italy; 13 Novartis Farma S.p.A., 21042 Origgio, Italy

**Keywords:** advanced breast cancer, MAP, MONALEESA-7, premenopausal, ribociclib, young breast cancer

## Abstract

Ribociclib plus an aromatase inhibitor and ovarian function suppression is the preferred first-line option for pre-/perimenopausal women with hormone receptor-positive/human epidermal growth factor receptor-2-negative advanced or metastatic breast cancer. We opened an italian managed access program (MAP) that permitted access to ribociclib to selected patients and allowed to collect informative results on the clinical impact of the therapy. The MAP (April 2018–May 2020) included 64 premenopausal patients, with characteristics similar to those of the MONALEESA-7 trial. Of 57 patients with a known response, 48 (84.2%) achieved a clinical benefit (i.e., complete response, N = 7 (12.3%); partial response, N = 17 (29.8%); stable disease, N = 24 (42.1%)), while 9 (15.8%) experienced tumor progression. Some patients (N = 15–23.4%) needed ribociclib dose reduction because of adverse events. Thereafter, the treatment was well tolerated, and no new safety signals emerged. Our study is the first reported Italian real-world evidence of ribociclib effectiveness in premenopausal HR+/HER2− advanced breast cancer patients. Response and clinical benefit rates were particularly encouraging compared with those of the ribociclib group of MONALEESA-7. Our work confirms that ribociclib in combination with endocrine therapy is highly effective in the treatment of premenopausal HR+/HER2− advanced breast cancer patients with an expected safety profile.

## 1. Introduction

Despite the large effort made by science and research, breast cancer is still the most common cancer worldwide. In Italy, it is the leading cause of cancer mortality among women [1]. In particular, it accounts for 41% of all cancer diagnoses and 28% of all cancer deaths in women aged <50 years, with a mean annual incidence increment of 1.6% observed between 2008 and 2016 [1].

Premenopausal women present a biologically distinct and more aggressive disease compared with postmenopausal patients [2,3], and experience poorer quality of life (QoL) [4] as they cope with premature menopause, emotional distress, and strain on their professional and personal lives [5,6]. 

Over 65% of breast cancers in women aged 50 years or younger are hormone receptor-positive (HR+)/human epidermal growth factor receptor-2-negative (HER2−) [7]. Standard first-line therapy for premenopausal women with HR+ advanced breast cancer (ABC) has traditionally consisted of ovarian suppression or ablation and hormone therapy [8]; however, endocrine therapy resistance and disease progression occur in most cases [9]. 

Ribociclib is an orally bioavailable, selective, small-molecule inhibitor of cyclin-dependent kinases (CDK) 4 and 6 [10]. Approval of ribociclib was granted in peri-/premenopausal patients based on the results of the MONALEESA-7 trial, the only phase 3, randomized clinical trial to assess a CDK4/6 inhibitor in combination with endocrine therapy and ovarian function suppression using goserelin, specifically designed for pre-/perimenopausal patients with HR+/HER2− ABC [11]. In the primary analysis, patients who received ribociclib plus endocrine therapy had a statistically significant and clinically meaningful improvement in progression-free survival (PFS) compared with those receiving placebo plus endocrine therapy. Median PFS was 23.8 months (95% confidence interval (CI) 19.2—not reached) in the ribociclib group compared with 13.0 months (11.0–16.4) in the placebo group, with a 45% reduction in the relative risk of progression (hazard ratio (HR) 0.55, 95% CI 0.44–0.69; *p* < 0.0001) [11]. The addition of ribociclib to endocrine therapy also yielded a significantly longer overall survival (OS) and statistically significant improvements in QoL compared with endocrine therapy alone [12,13,14]. In particular, after a median follow-up of 53.5 months (46.9–66.4 months), the median OS was 58.7 months in the ribociclib group and 48.0 months in the placebo group (HR, 0.76, 95% CI 0.61–0.96), with a 24% relative reduction in the risk of death with ribociclib [14]. Notably, this is the longest median OS reported in pre-/perimenopausal women with HR+/HER2− ABC treated in first line. The most common adverse reactions (incidence ≥20%) were neutropenia, nausea, infections, fatigue, diarrhea, leukopenia, vomiting, alopecia, headache, constipation, rash, and cough. Most adverse events (other than neutropenia) were grade 1 or 2 and manageable with ribociclib dose adjustments; few led to treatment discontinuation (4% of patients) [11].

To provide access to ribociclib before its commercial availability and reimbursement in Italy, we opened a managed access program (MAP), which allowed to collect informative efficacy and safety data in a real-world setting. Here, we report the outcomes of premenopausal HR+/HER2− ABC women treated with ribociclib in combination with a non-steroidal aromatase inhibitor (NSAI) and a luteinizing hormone-releasing hormone (LHRH) agonist within the MAP.

## 2. Materials and Methods

The Italian ribociclib MAP ran in 22 sites from April 2018 to May 2020, until ribociclib became commercially available and reimbursable for premenopausal patients in the country. Patients were granted access to the MAP after the Novartis medical team had reviewed the unsolicited request forms received from treating physicians. The MAP included premenopausal or perimenopausal adult women with confirmed HR+/HER2− locally advanced or metastatic breast cancer. Menopausal status was defined according to MONALEESA 7 protocol [11]. Patients who had received (neo)adjuvant endocrine therapy for breast cancer were eligible 12 months after the last dose of adjuvant NSAI treatment, or if tamoxifen was the last prior (neo)adjuvant therapy and the last dose had been given <12 months prior to starting treatment with ribociclib. Only patients with Eastern Cooperative Oncology Group performance status 0–2 and adequate bone marrow and organ functions have been enrolled in the study.

Premenopausal women who had received prior treatment with any CDK4/6 inhibitor or more than one line of prior chemotherapy for ABC were not eligible for the program. Other key exclusion criteria were the presence of symptomatic visceral disease and cardiac disease or a history of cardiac dysfunction, including QT interval (QTc) >450 ms at baseline.

The program was approved by the Italian Local Health Authority, and each site’s ethics committee approved the requests for each patient. Specific written informed consent was obtained from each patient prior to the start of treatment.

### 2.1. Treatment

Patients were treated with ribociclib in combination with an NSAI and an LHRH agonist. Ribociclib was administered orally, 600 mg (3 × 200 mg tablets) once a day for 21 consecutive days followed by a 7-day break (i.e., 28-day cycle). Dose adjustments were permitted for patients who did not tolerate the dosing schedule specified in the treatment plan (guidelines for dose adjustments according to the ribociclib data sheet) [10]. The NSAI and LHRH agonist were used at standard doses. Use of tamoxifen was not allowed because of the higher incidence of QTc prolongation when combined with ribociclib compared with NSAI [10,11].

Patients received the treatment until disease progression, symptomatic deterioration, unacceptable toxicity, death, withdrawal of consent, or commercial availability of ribociclib, whichever occurred first.

### 2.2. Assessments, Data Collection, and Statistical Analysis

Visits were recommended on days 1 and 15 of cycles 1 and 2, and on day 1 of subsequent cycles. Participating physicians were asked to report a pseudonymized clinical data set for each patient including medical history, drug dosage reduction and reasons for reduction, tumor response, and treatments after progression. Safety information (adverse events) was reported according to local regulatory requirements.

All patients who signed the informed consent form and took at least one dose of treatment (treated patients) were included in the statistical analysis, which is descriptive only. We present baseline characteristics as proportions for categorical variables and mean ± standard deviation (SD) or median and range for continuous variables, and describe the rate of best response and clinical benefit considering treated patients with available data. We also provide the proportion of patients with first ribociclib dose reduction (from 600 mg to 400 mg) together with the time to first reduction, as well as the proportion of patients who discontinued treatment together with reasons for treatment discontinuation. Finally, a description of the first post-progression treatment is reported for patients who discontinued treatment because of progressive disease.

## 3. Results

Of the 67 patients who were granted access to the MAP, 64 received at least one dose of ribociclib and were included in this analysis. When the MAP was closed in May 2020 because of local reimbursement of the drug, treatment was still ongoing for 38 patients (59.4%), whereas 26 patients (40.6%) had discontinued treatment because of disease progression (N = 15, 57.7%), loss of follow-up (N = 2, 7.7%), adverse events (N = 3, 11.5%, specifically QTc prolongation, hepatic toxicity, and persistent haematological toxicity), or unknown reasons (N = 6, 23.1%). In particular, three women discontinued therapy for an adverse event. One of these patients showed a worsening transaminitis. She was in stable disease (SD) when ribociclib was discontinued and continued treatment with letrozole alone for one year, when bone-disease progression occurred. Another patient discontinued for persistent grade 3 haematological toxicity (platelet count: 40,000/µL), then received exemestane monotherapy until hepatic progression, which occurred 4 months after ribociclib discontinuation. Finally, an increase from 440 msec to 492 msec in the QTc interval was reported in one patient. The QTc value was 484 msec after 14 days. At that time, it was the decision of the physician to discontinue ribociclib therapy. 

Patients had a median age of 46.7 (range: 31–57) years, 62 patients (96.9%) had stage IV cancer and 2 (3.1%) had stage III not amenable to curative surgery; 33 (51.6%) patients were newly diagnosed with metastatic disease and 48.4% experienced a recurrence after or while on adjuvant treatment. Among patients with recurrence, 21 (35.0%) had received chemotherapy followed by endocrine therapy as adjuvant therapy, 5 (8.3%) had received endocrine therapy alone (tamoxifen or tamoxifen + LHRH agonist), and 1 patient (1.7%) had received chemotherapy only. Four patients had missing data for adjuvant therapy. In the metastatic setting, four (6.3%) had received first-line chemotherapy. The number of patients with visceral metastases was 29 (45.3%) and the number of those with non-visceral metastases was 35 (54.7%); specifically, 22 had bone only disease, 5 had lymph nodes only disease, and 8 had both bone and lymph nodes metastasis (Table 1). 

A clinical benefit (complete response (CR), partial response (PR), or stable disease (SD)) was achieved in 48 patients (84.2%)—out of 57 for which the response is known; the best responses are detailed in Table 2. Nine (15.8%) patients experienced progression, while for a further seven patients, the response was unknown (clinicians did not have to provide data, as MAP is not a clinical trial). 

Dosage was reduced from 600 mg to 400 mg for 15 patients (23.4%) owing to adverse events, with a mean time to dose reduction of 4.34 ± 2.01 months (range: 1.5–7.9). Only one patient required dose reduction from 400 mg to 200 mg.

Of the 15 patients who discontinued treatment owing to disease progression (including the 9 for which the radiological response is known, and those for which the progression was adjudicated by the clinician), all but one received an active treatment afterward: 6 (42.9%) chemotherapy, 1 (7.1%) endocrine therapy, 3 (21.4%) everolimus + exemestane, and 4 (28.6%) unknown therapy (Table 3).

## 4. Discussion

The MONALEESA 7 trial has clearly proven the efficacy and safety of ribociclib in pre-/perimenopausal women [11]. It has happened before that the highly selected population of clinical trials has failed to accurately predict the clinical impact of new therapies, and physicians often look to real world data as a blueprint of what to expect in daily practice. Here, we present the data from the Italian ribociclib MAP, with a population largely overlapping that of the MONALEESA 7 trial, but at the same time, more representative of the southern European clinical landscape, also allowing ECOG PS 2 patients, and with a slightly older median age. This is the first real-world data report in the pre-/perimenopausal setting. Apart from the population, the main difference between MONALEESA-7 and this MAP is that, in the randomized trial, the endocrine therapy partner was chosen based on previous adjuvant or neoadjuvant treatment or on investigator or patient preference, while in our MAP, ribociclib was administered in combination with an AI and LHRH agonist, but not with tamoxifen, respecting the actual clinical indication. Moreover, while in the MONALEESA-7 trial, the proportion of patients with de novo advanced/metastatic disease at diagnosis was 41% [11], in the ribociclib MAP as much as 51.6% presented with de novo metastatic breast cancer.

Response rates were particularly encouraging when compared with those of the ribociclib cohort of the MONALEESA-7 trial [11]: 12% of patients had a CR in the MAP compared with 2% in the MONALEESA-7 trial. PR rates were 29% and 39%; SD rates were 42% and 32%; and clinical benefit rates (CR + PR + SD) were 84% and 79%, respectively. Treatment was generally well tolerated, and treatment-related adverse events were manageable by temporary discontinuations, dose reduction, and/or standard medical therapy. The adverse events were consistent with the known safety profile of ribociclib when administered with an NSAI [11]. There were no unexpected major safety findings in this population. The Italian ribociclib MAP is limited by not being a clinical trial, thus not providing data on survival and HRQoL. Nevertheless, these results are worth sharing as they confirm the safety and efficacy of ribociclib, outside of clinical trials, in pre-/perimenopausal women in daily clinical practice.

## 5. Conclusions

We conducted a MAP study to provide early access to therapy for a cohort of cancer patients without other therapeutic options. Despite the limitation inherent to the type of our study, the data collected in our program support a safe use of ribociclib in clinical practice for the treatment of pre-/perimenopausal women with HR+/HER2− advanced or metastatic breast cancer.

## Figures and Tables

**Table 1:** Baseline patient and disease characteristics. All data are presented as frequencies (N (%)) unless specified.

	Total N = 64
**Age (years), median (range)**	46.7 (31–57)
˂40 years	9 (14.1)
≥40 years	55 (85.9)
**Disease stage at initial diagnosis**	
Stage I	5 (7.8)
Stage II	13 (20.3)
Stage III	14 (21.9)
Stage IV	32 (50.0)
**Current disease stage**	
Stage III	2 (3.1)
Stage IV	62 (96.9)
**Metastatic disease status**	
Recurrent	31 (48.4)
De novo	33 (51.6)
**Metastatic sites**	
Visceral	29 (45.3)
Non-visceral	35 (54.7)
Bone only	22 (34.4)
Lymph nodes only	5 (7.8)
Bone + lymph nodes	8 (12.5)
Prior antineoplastic adjuvant therapy *	
No prior adjuvant therapy	33 (55.0)
Endocrine therapy only	5 (8.3)
Tamoxifen	2 (3.3)
Tamoxifen + LHRH agonist	3 (5.0)
Chemotherapy only	1 (1.7)
Chemotherapy followed by endocrine therapy	21 (35.0)
**Prior chemoterapy in metastatic setting**	4 (6.3)

* Four unknown cases were reported, but not included in this table; they were not considered in the percentage calculation for prior antineoplastic adjuvant therapy. LHRH, luteinizing hormone-releasing hormone.

**Table 2:** Best response. All data are presented as frequencies (N (%)) unless specified.

	Total N = 57
**Best response ^1^**	
Complete response	7 (12.3)
Partial response	17 (29.8)
Stable disease	24 (42.1)
Progressive disease	9 (15.8)

^1^ Percentages computed on patients who had information about best response. Clinical benefit achieved if patient had a complete response or partial response or stable disease.

**Table 3:** First post-progression treatment. All data are presented as frequencies (N (%)) unless specified.

	Total N = 64
**Patients with progressive disease**	15 (23.4)
**Patients who received post-progression treatment ^1^**	
No	1 (6.7)
Yes	14 (93.3)
**First post-progression treatment ^2^**	
Chemotherapy	6 (42.9)
Hormonotherapy	1 (7.1)
Everolimus + exemestane	3 (21.4)
Unknown	4 (28.6)

^1^ Percentages computed on patients with progressive disease. ^2^ Percentages computed on patients with progressive disease who received post-progression treatment.

## Data Availability

The data presented in this study are available on request to the corresponding author.
